# Microcystin-Induced Immunotoxicity in Fishes: A Scoping Review

**DOI:** 10.3390/toxins13110765

**Published:** 2021-10-29

**Authors:** Wang Lin, Tien-Chieh Hung, Tomofumi Kurobe, Yi Wang, Pinhong Yang

**Affiliations:** 1 Hunan Provincial Collaborative Innovation Center for Efficient and Health Production of Fisheries, Hunan Provincial Key Laboratory for Health Aquaculture and Product Processing in Dongting Lake Area, Hunan Provincial Key Laboratory for Molecular Immunity Technology of Aquatic Animal Diseases, Hunan Engineering Research Center of Aquatic Organism Resources and Environmental Ecology, Zoology Key Laboratory of Hunan Higher Education, College of Life and Environmental Sciences, Hunan University of Arts and Science, Changde 415000, China; linwang@huas.edu.cn; 2 Department of Biological and Agricultural Engineering, University of California, Davis, CA 95616, USA; thung@ucdavis.edu (T.-C.H.); yyiwang@ucdavis.edu (Y.W.); 3 Department of Fisheries Resources and Environment, College of Fisheries, Huazhong Agricultural University, Wuhan 430070, China; 4 Department of Anatomy, Physiology, and Cell Biology, University of California, Davis, CA 95616, USA; tkurobe@ucdavis.edu

**Keywords:** microcystins, neuroendocrine-immune network, inflammatory responses, immunotoxicity, fish

## Abstract

Cyanobacteria (blue-green algae) have been present on Earth for over 2 billion years, and can produce a variety of bioactive molecules, such as cyanotoxins. Microcystins (MCs), the most frequently detected cyanotoxins, pose a threat to the aquatic environment and to human health. The classic toxic mechanism of MCs is the inhibition of the protein phosphatases 1 and 2A (PP1 and PP2A). Immunity is known as one of the most important physiological functions in the neuroendocrine-immune network to prevent infections and maintain internal homoeostasis in fish. The present review aimed to summarize existing papers, elaborate on the MC-induced immunotoxicity in fish, and put forward some suggestions for future research. The immunomodulatory effects of MCs in fish depend on the exposure concentrations, doses, time, and routes of exposure. Previous field and laboratory studies provided strong evidence of the associations between MC-induced immunotoxicity and fish death. In our review, we summarized that the immunotoxicity of MCs is primarily characterized by the inhibition of PP1 and PP2A, oxidative stress, immune cell damage, and inflammation, as well as apoptosis. The advances in fish immunoreaction upon encountering MCs will benefit the monitoring and prediction of fish health, helping to achieve an ecotoxicological goal and to ensure the sustainability of species. Future studies concerning MC-induced immunotoxicity should focus on adaptive immunity, the hormesis phenomenon and the synergistic effects of aquatic microbial pathogens.

## 1. Introduction

**Figure 1:**
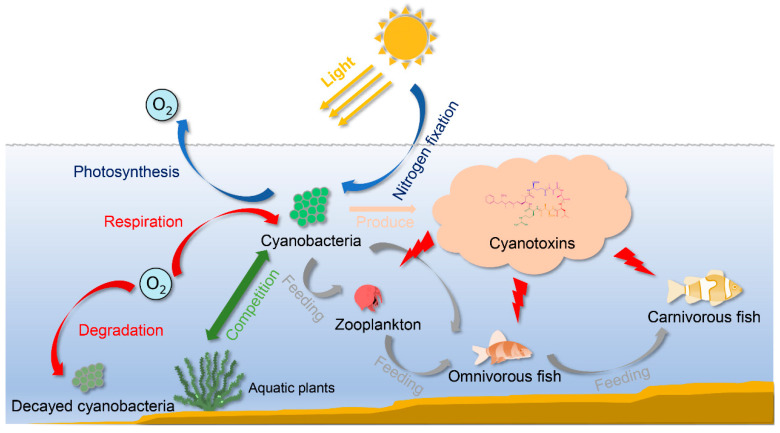
Impact of cyanobacteria and cyanotoxins on the aquatic food web. The respiration and degradation of CyanoHABs result in oxygen deprivation in the water. The cyanotoxins, produced and released by cyanobacteria, can harm aquatic animals through biological concentration or food chain transfer.

Cyanobacteria are the oldest known oxygenic photoautotroph on earth. The proliferation of cyanobacteria between 2.45 and 2.32 billion years ago greatly changed the foregoing anoxic biosphere by producing oxygen and accelerating the evolution of higher flora and fauna [1,2]. Many genera of cyanobacteria can regulate atmospheric nitrogen and store phosphorus, which enables them to adapt to multiple geographical conditions, including aquatic and terrestrial environments [3,4]. However, the aquatic environmental and hydrological alterations caused by anthropogenic activities have given rise to a series of serious environmental problems [5,6]. In recent decades, the frequency of harmful algal blooms (CyanoHABs) increased due to eutrophication and climate changes [7]. The deleterious outcomes of these blooms cause the loss of water clarity, which negatively affects aquatic plants, phytoplankton, and zooplankton [8,9]. The decomposition of CyanoHABs can also result in oxygen depletion, and subsequently lead to fish death [10]. A variety of cyanobacterial species also produce toxic secondary metabolites (cyanotoxins) during cell lysis associated with natural aging or environmental stress, which can greatly induce toxic effects in aquatic food webs (Figure 1).

**Figure 2:**
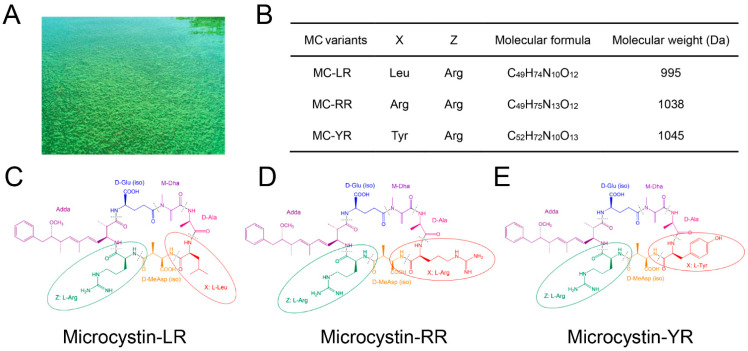
Characteristics of cyanobacterial blooms and microcystins: (**A**) cyanobacteria blooms in the San Francisco Estuary (photo provided by Dr. Peggy Lehman, California Department of Water Resources); (**B**) molecular formula and molecular weight of microcystin variants; (**C**) microcystin-LR, with the amino acid leucine (L) and arginine (R); (**D**) microcystin-RR, with the amino acid arginine (R) and arginine (R) and (**E**) Microcystin-YR, with the amino acid tyrosine (Y) and arginine (R).

Among various classes of cyanotoxins, MCs are the most widespread in freshwater environments. Until now, over 270 different MC congeners have been determined, and Microcystin-LR (MC-LR), Microcystin-RR (MC-RR), and Microcystin-YR (MC-YR) are the three of most concern and widely studied [11,12,13]. The molecular formula and molecular weight of the three commonly distributed MCs are shown in Figure 2. MC-LR is the most toxic and abundant structural congener in freshwater, followed by MC-RR and MC-YR [14,15]. As one of the most toxic and commonly distributed variants, MC-LR is a great worldwide concern due to its acute poisonous lethal effects and chronic carcinogenesis [16,17]. According to the guidelines of the World Health Organization (WHO), the maximum concentration of MC-LR should not exceed 1 μg/L in drinking water [18] in order to be safe; the MC-LR concentrations in natural waters are normally within the scope of 0.1 to 10 μg/L [19]. Previous studies have demonstrated that it is difficult to degrade MC-LR in water during an algal bloom, which could greatly impact the normal function of aquatic organisms and terrestrial organisms [20,21,22,23,24].

The inhibition of protein phosphatase 1 and 2A is the classic toxic mechanism of MCs, tightly associated with their cytotoxicity and tumorigenesis [25,26,27]. Several studies have demonstrated that oxidative stress plays a vital role in the MC-induced hepatotoxicity, mainly characterized by the overproduction of reactive oxygen species (ROS) and the depletion of glutathione [28]. In order to alleviate the negative effects from oxidative stress, an antioxidant system developed in aquatic organisms. This system produces antioxidant substances, including enzymatic components such as catalase (CAT), superoxide dismutase (SOD), glutathione peroxidase (GPx), and non-enzymatic components, such as glutathione (GSH) [29].

Increasing evidence has shown that MCs mainly accumulate in the liver and cause hepatotoxicity, but immune organs, such as the spleen and head kidney, are also vulnerable to MCs exposure [30,31,32]. Many studies have found that MCs can exert toxic effects on lymphocytes as well as phagocytes, subsequently resulting in immune dysfunction. In an in vitro study, Rymuszka et al. [33] suggested that the proliferation of isolated splenic lymphocytes of rainbow trout (*Oncorhynchus mykiss*) exhibited a significant decrease in the 40 mg/mL MC- LR group while a significant increase in the 1 mg/mL MC-LR group. In an in vivo experiment, Wei et al. [34] found a significant downregulation of the immune-related genes in the spleen and head kidney when grass carp (*Ctenopharyngodon idella*) were injected with 50 μg/kg MC-LR. Hence, MCs were proven to disturb fish immune function, which could increase their susceptibility to pathogens and eventually result in fish death.

The immune system is a collaborative network of immunological molecules, immune cells, and lymphoid organs, and is hypersensitive to toxic substances [35]. It is thought that MCs can enter the immune organs and cause immunotoxicity [36]. However, the details of the mechanism of MC-induced immunotoxicity in fish are still incomplete. The aim of this scoping review is to organize the existing works involving the immunotoxicity of MCs in fish and to summarize the potential mechanism of MC-induced immune dysfunction. The present literature review was conducted using PubMed (https://pubmed.ncbi.nlm.nih.gov/ (accessed on 26 October 2021)) with the goal of identifying related articles.

## 2. Immune System of Fish

The fish immune system is divided into innate (non-specific) immunity and adaptive (specific) immunity. Innate immunity substantially acts as the hosts’ first line of defense against external pathogens, while specific immunity exerts a key role to prevent recurrent infections by generating membrane-bound receptors and memory cells [37]. Fish are known as the earliest vertebrates with both specific and non-specific immunity, though their adaptive immunity is not as developed as the higher vertebrates [38,39]. The innate immunity to antigens relies on the pattern recognition receptors (PRRs), which recognize danger-associated molecular patterns (DAMPs), or pathogen-associated molecular patterns (PAMPs) [40]. Numerous PRRs have been extensively studied, including the Toll-like pattern recognition receptors (TLRs), RIG-I-like receptors (RLRs), and Nod-like pattern recognition receptors (NLRs) [41,42]. The host’s PPRs activate immune signaling pathways when they recognize the conserved structure of PAMPs, subsequently resulting in an inflammatory response [43,44].

The fish immune organs include the thymus, head kidney, spleen, and mucosa-associated lymphoid tissue, which provide proper sites for immune cells to differentiate and proliferate [45]. The thymus is the main site of differentiation and maturation of the T lymphocytes, which are mainly responsible for cellular immune function [46]. The head kidney is responsible for hematopoietic and immune functions in fish [37,47]. The spleen is an important site for the production and maturation of various erythrocytes and granulocytes [48]. The mucosa-associated lymphoid tissue of fish mainly consists of the skin-, gill-, and gut-associated lymphoid tissue, which can induce a local immune response and function [49].

Fish immune cells, including lymphocytes and phagocytes, mainly distribute in the blood, lymphatic fluid, and immune organs [50,51]. T cells are responsible for mediating cellular immunity, while B cells are involved in the synthesis of antibodies in humoral immunity [52,53]. Zwollo et al. [54] found that the maturation, metastasis, and response of B cells in bony fish are similar to those of mammals. The phagocytes of fish mainly include monocytes, macrophages, and granulocytes, which can phagocytize senile cells and foreign bodies through deformation movement [55,56].

A variety of immune molecules including cytokines, lysozymes, and the complement system also play an important role in the immune function of fish [57,58,59]. Lysozyme is widely found in the macrophage, serum, and mucus, and can destroy Gram-positive bacteria cells [60]. Kong et al. [61] found that the lysozyme activity exhibited a significant increase when fish were exposed to infectious bacteria or other stress factors. The complement system, which plays a vital role in clearing the potential pathogens in the host, can activate various immune function processes, like phagocytosis, pathogen cytolysis and inflammation [62,63,64]. The activation of the complement system consists of the classical pathway, lection pathway and alternative pathway, which are responsible for the target identification and protease complex formation for the complement component C3 activation [65].

## 3. Immunotoxicity of MCs on Fish

### 3.1. In Vivo Studies

#### 3.1.1. Cyanobacteria Cells

As shown in Table 1, a number of studies were dedicated to discussing the immunotoxicity of cyanobacterial cells on fish. Atencio et al. [66] found that the levels of lipid peroxidation (LPO), GR, and GPx exhibited a significant increase in kidney and liver of tilapia (*Oreochromis niloticus*) when exposed to an oral dose of 120 μg MC-LR/fish for 24 h, while CAT, SOD and GST showed a significant decrease. In a long-term study, crucian carp (*Carassius auratus*) were fed diets containing 20% and 40% of cyanobacteria lyophilized powder made from *Microcystis* (equivalent to 1.41 mg/g MCs dry weight) and collected from Lake Dianchi (Yunnan, China) [67]. After an exposure of 30 days, splenic hyperemia and hemorrhages were observed in the 40% group. Lysozyme activity showed a significant increase in the 20% group, but a significant decrease in the 40% group, indicating that fish immunity exhibited immunostimulation at low MCs doses, but was immunosuppressive at high MC doses. In another study, blunt snout bream (*Megalobrama amblycephala*) were fed diets containing 15% and 30% of cyanobacteria lyophilized powder for 30 d [68]. After the exposure, the edematous mitochondria of head kidney lymphocytes, as well as a decreased phagocytosis activity of macrophages, were observed.

In addition to oral feeding, immersion is also utilized to investigate the immunotoxicity on fish. Hematological indices are reliable indicators to evaluate the immune function. In an acute study, Kopp and Heteša [69] suggested that various blood indices like total protein (TP), aspartate aminotransferase (AST), alanine aminotransferase (ALT), and lactic dehydrogenase (LDH) exhibited a remarkable increase in juvenile carp when exposed to cyanobacteria blooms. Kopp et al. [70] demonstrated that numerous plasma indicators including albumin (ALB), cholesterol (CHOL), glucose (GLU), creatinine (CRE), alkaline phosphatase (ALP), and TP showed a significant decrease when silver carp (*Hypophthalmichthys molitrix*) were exposed to cyanobacteria cells containing 2.8–4.7 μg MCs. In a long-term field study, silver carp were collected monthly during a *Microcystis* bloom period in Lake Taihu, and MC contents were detected in the fish kidney [71]. The nephritic ultrastructural damages were mainly characterized by the dilation of bowman’s space and lysosomes proliferation. The nephritic antioxidant enzyme activities exhibited a dramatic increase, implying the antioxidant system was activated to alleviate oxidative stress.

#### 3.1.2. Cyanobacteria Extracts

The in vivo studies of cyanobacterial extracts-induced immunotoxicity on fish are summarized in Table 1. In an acute study, healthy crucian carp were intraperitoneally (IP) injected with cyanobacterial extracts containing 50 and 200 μg/kg MC-LR, and plasma biochemical parameters including LDH, ALP, AST, and ALT showed a significant increase [72]. Rymuszka and Adaszek [73] reported that cyanobacteria extract can cause ROS overproduction, as well as lymphocytes proliferation inhibition in the blood and head kidney of common carp (*Cyprinus carpio* L.). The upregulation of tumor necrosis factor alpha (TNF-α), interleukin 1 beta (IL-1β), and interleukin 10 (IL-10) gene expression were observed when exposed to an MC-containing extract. In a sub-chronic study, Palíková et al. [74] reported that common carp larvae exposed to the cyanobacteria crude extracts (equivalent to 1.3 μg/L or 13 μg/L MCs) for 30 d, the hemoglobin concentrations and hematocrit values showed a significant increase while the phagocytic activities of leukocyte exhibited a significant decrease.

#### 3.1.3. Pure Microcystins

The in vivo studies of pure microcystin-induced immunotoxicity on fish are summarized in Table 2. In an acute study, a significant increase in serum ALT, AST, lysozyme activity, complement C3, and the cytokines contents including TNFα, IL1β, and IFNγ were observed when silver carp intraperitoneally injected with MC-LR [75]. In a chronic study, Chen et al. [76] demonstrated that IP injection of 150 μg/kg MC-LR can result in a significant decrease in serum complement C3 and lysozyme activity. However, antioxidant indexes like CAT, SOD, GPx, and LPO levels were remarkably increased, suggesting the antioxidant system was activated in the common carp. MCs exposure can also induce changes in immune parameters in organs, except alterations in hematological biochemical indicators. Grass carp were IP injected with 50 μg/kg/d MC-LR, and the spleen and head kidney were sampled at 1, 2, 7, 14, and 21 d [34]. After exposure, the chromatin compaction, mitochondria edema, and apoptotic lymphocytes were found in the spleen and head kidney. In another study, Li et al. [77] found MC-LR could significantly upregulate the mRNA levels of interleukin-8 (*il8*), a classic inflammatory gene, in bighead carp (*Aristichthys nobilis*) in a dose-dependent manner. Similarly, grass carp were IP injected with 25, 75, and 100 μg/kg MC-LR [78]. Transcription levels of cytokine signaling 3 (*soc3*) and heat shock protein 70 (*hsp70*) showed a significant increase after 96 h of exposure, indicating an activation of the defense system. The oxidant-antioxidant balance is vital for immune function in fish. In an acute study, Okogwu et al. [79] reported that IP injection of MC-RR at 50 and 200 μg/kg MC-LR for 48 h could significantly decrease the nephritic total antioxidant capacity (T-AOC) and GPx activities in gold fish (*Carassius auratus*). Similarly, Prieto et al. [80] demonstrated that the increased antioxidant function was observed in tilapia kidney when fish were IP injected with 500 μg/kg MC-RR for 7 d, which indicated that the antioxidant system plays a vital role in the protection of fish health.

For immersion cases, Li et al. [81] demonstrated that acute exposure of 800 μg/L MC-LR could interfere with lymphocytes deferrization and TCR/ig arrangement in zebrafish (*Danio rerio*) larvae by upregulating early lymphoid development genes, including recombination activation gene-1 (Rag1), recombination activation gene-2 (Rag2), Ikaros, GATA1, Lck, and T-cell receptor-α (TCRα). In a sub-chronic study, nucleus deformation and edematous mitochondria were noticed in the spleen when zebrafish were exposed to 20 μg/L MC-LR [82]. The mRNA levels of splenic interferon-1 (*ifn1*), *il8*, interleukin-1β (*il1β*), and tumor necrosis factor-α (*tnfα*) exhibited a significant increase in the 1 and 20 μg/L groups. In our previous study, male zebrafish were exposed to environmentally relevant concentrations of MC-LR for 30 d. Lin et al. [31] found that the number of melano-macrophage centers and serum complement C3 levels and splenic complement component C3b (*c3b*) expression showed a significant increase in the low MC-LR exposure groups (0.3, 1, and 3 μg/L). However, the degeneration of splenic macrophages and lymphocytes, and the remarkable decrease in C3 levels were observed in the high MC-LR exposure groups (10 and 30 μg/L). These findings indicated MC-LR exhibits diverse influence on the fish immune system, which is characterized by an inflammatory activation in the low MC-LR exposure groups, but immune inhibition in the high MC-LR exposure groups. Lin et al. [83] found chronic inflammation and immune disorders induced by MC-LR may be mediated through the myeloid differential protein-88-dependent Toll-like receptor (TLR/MyD88) signaling pathway. These findings indicated that MCs could cause varied influences on the antioxidant system and the innate immune system in fish, which may greatly depend on the exposure routes and concentrations/doses. Furthermore, Falfushynska et al. [84] demonstrated that 20 μg/L MC-LR could induce multifaceted toxic effects including oxidative stress and apoptosis in zebrafish, which characterized by the increase in thiobarbituric acid-reactive substances (TBARS) levels and caspase-3a (*cas3a*) and caspase-3b (*cas3b*).

### 3.2. In Vitro Studies

The in vitro studies of pure microcystins induced immunotoxicity on fish are summarized in Table 3. In an acute study, Zhang et al. [85] assessed the induction of apoptosis by environmentally relevant levels of MC-LR and MC-RR on crucian carp lymphocytes. Apoptotic features characterized by apoptotic bodies formation and nuclear chromatin condensation were observed in the 1, 5, and 10 nM MC-LR or MC-RR groups, which indicated that MCs could result in lymphocytes apoptosis. Zhang et al. [86] demonstrated MC-induced apoptosis was tightly associated with ATP depletion and ROS overproduction, which implied that mitochondria and oxidative stress play a vital role in MC-induced toxicity on crucian carp lymphocytes. In another study, Zhang et al. [87] found that 10 nM MC-RR could result in ATP depletion and ROS elevation, as well as mitochondrial membrane potential disruption in crucian carp lymphocytes. These findings revealed that the alteration of intracellular Ca^2+^, ROS, mitochondrial membrane potential, and ATP are closely associated with MC-induced apoptosis. Similarly, Zhang et al. [88] demonstrated that the apoptotic pathway was stimulated, and antioxidant defense system were impaired in crucian carp lymphocytes under MC-LR exposure, which confirmed that apoptosis always come along with oxidative stress.

To investigate the effects of MC-LR on the cell proliferation, rainbow trout lymphocytes were isolated from immune organs and peripheral blood, then exposed to 1, 5, 10, 20, and 40 mg/mL MC-LR [33]. Lymphocytes’ proliferation showed a significant increase in the 1 mg/mL MC-LR group but a significant decrease in the 40 mg/mL MC-LR group, which implied that the applied doses caused different immunomodulatory effects. In another study, phagocytic cells were separated from the blood of rainbow trout and exposed to 1, 5, 10, and 20 μg/mL MC-LR [89]. The results indicated that MC-LR could decrease phagocytic cell numbers in a concentration- and time-dependent pattern, and a possible reason for such cytotoxic activity might be associated with oxidative stress. ROS overproduction and GSH reduction in *C. idellus* kidney (CIK) cells, as well as a reduced cell viability, were observed when exposed to 100 μg/L MC-LR for 48 h, which indicated that the oxidative stress and cytoskeletal disruption may have had a mutual effect on inducing renal toxicity [90].

Head kidney cells from omnivorous carp were isolated and exposed to 0.01, 0.1, 0.5, and 1 μg/mL pure MC-LR to evaluate the possible effects of MC-LR on their immune functions [91]. The phagocytes’ metabolic activity exhibited an inhibitory effect in the high MC-LR group, while a stimulatory effect in the low MC-LR group was observed. These results suggested that the increased MC-LR concentrations could disturb the immunologic homeostasis of the fish body and subsequently lead to an elevated susceptibility. Moreover, Rymuszka et al. [92] demonstrated that MC-LR induced apoptosis and necrosis in common carp lymphocytes. In another study, blood and head kidney leukocytes were isolated from carp, then exposed to 0.01 and 0.1 μg/mL MC-LR for 4 h [93]. Pro-inflammatory cytokines (*tnfα* and *il1β*) showed a significant downregulation, while anti-inflammatory cytokines (*il10* and *tgfβ*) showed a significant upregulation. This indicated that acute MC-LR exposure could inhibit an inflammatory response.

## 4. Potential Mechanism of MC-Induced Immunotoxicity

An increasing number of studies were conducted to explore the mechanism of MC-induced immunotoxicity on fish. Aside from the classic toxic mechanism of PP1/2A inhibition, other physiological processes including oxidative stress, inflammatory responses, and apoptosis also arouse public concern. We summarized all possible mechanisms to provide a comprehensive understanding of how MCs enter fish organs and how they disrupt the fish immune system.

### 4.1. Adsorption and Accumulation of MCs

In order to exert toxic effects, sufficient concentrations of MCs must enter specific organs, tissues, or cells [27,94]. Due to the high molecular weight of MCs, their uptake and accumulation in fish occurs through active transport [95]. Both field and laboratory experiments have proven that MCs can accumulate in the spleen, and then interfere with the immune function [36,96]. Organic anion transporting polypeptides (OATP) play a critical role in the active transmembrane transport of MCs [97,98]. MC-LR is identified as a hepatotoxin due to its high expression of hepatocyte-specific OATP carriers [99]. The differential expression of Oatp subtypes results in a varying tissue distribution of MCs in fish. It was demonstrated that the expression of zebrafish Oatp subtypes in diverse organs is tightly associated with its specific toxicity, and the Oatp1f was only expressed in the zebrafish kidney [100]. However, very few studies focus on the OATP subtypes in fish spleen or head kidney.

### 4.2. Inhibition of PP1 and PP2A

Protein phosphorylation, catalyzed by protein phosphatases (PPs), is one of the most important dynamic processes associated with cellular homeostasis. PP1 and PP2A are major protein phosphatases in cells that are involved in the cytoskeleton dynamic, cell mobility, cell proliferation, and cell death [101,102]. Liang et al. [103] demonstrated that MCs could not only inhibit enzyme activity via regulating protein phosphatase activity, but also affect protein expression directly. The alterations of PP1/PP2A activities are tightly associated with cellular cytotoxicity and cytoskeletal disruption caused by MCs, which reveal the importance of protein phosphatases in MC-induced immunotoxicity [104,105].

### 4.3. Oxidative Stress

Oxidative stress, mainly caused by ROS overproduction, is a general toxicity mechanism of various xenobiotics or chemicals including MCs [106,107]. The antioxidant enzyme system is usually activated to alleviate the oxidative stress. Malbrouck and Kestemont [96] demonstrated that the alterations of antioxidant enzymes activities were greatly dependent on MCs exposure concentrations and exposure routes. Evidence has shown that both pure MCs and cyanobacterial cells can cause ROS overproduction, subsequently interfering with antioxidant enzyme activities, and the induction of lipid peroxidation in fish [108]. Oxidative stress induced by environmental pollutants is usually associated with the immune function of organisms [109,110]. It is well known that fish largely rely on the nonspecific immune system, which is tightly linked to the oxidant–antioxidant balance of the immune organs. Our previous study demonstrated that 30 μg/L of pure MC-LR resulted in a significant decrease in splenic GSH content in zebrafish [111]. GSH could exert a detoxification effect by binding MCs to form MC-GSH conjugates, and the reduction in cytosol GSH implied an excessive ROS production [112,113].

### 4.4. Immune Cell Damages

Several studies suggest that MC-LR can accumulate in the spleen and result in pathological lesions and immune dysfunction in various fish species such as whitefish (*Coregonus lavaretus*), trout, and crucian carp [67,114,115,116]. Rymuszka et al. [33] suggested that the rainbow trout lymphocyte viability showed a dose-dependent decrease. Wei et al. [34] demonstrated that MC-LR caused damage to the mitochondria of the splenic lymphocytes of grass carp when IP injected with MC-LR. Those results revealed that the MC-induced immunomodulatory effects on the proliferation and viability of fish lymphocytes rely on the different exposure concentrations and the target species. Apart from lymphocytes, Neumann et al. [117] demonstrated that phagocytes, including neutrophils and macrophages, could limit the transmission of an infectious source and destroy the phagocytosed pathogens. Research showed that MC-LR exposure could suppress the phagocytic ability of erythrocytes in juvenile common carp and silver carp [74,118]. Sierosławska et al. [89] found that the phagocytosis of isolated phagocytic cells from rainbow trout exhibited a significant increase when exposed to MC-LR. In our previous study, Lin et al. [31] suggested that splenic ultra-pathological alterations were observed when exposed to various MC-LR concentrations. Specifically, the formation of melano-macrophage centers and lymphocyte pseudopods was observed in the low MC-LR groups, while the degeneration of macrophages and lymphocytes was observed in the high MC-LR groups. Such evidence suggests that the MCs exposure could result in immunotoxicity by damaging the structure of the immune cells.

### 4.5. Inflammation

Immune cells are activated and produce cytokines when induced by various pathogens, exogenous chemicals, or environmental stress [119]. Cytokines, including TNFs, ILs, and IFNs, are mainly secreted by lymphocytes, macrophages, and granulocytes [120]. The cytokines in fish are similar to those in mammals, and several homologues have been cloned and investigated [121,122,123]. TNFα and IL1β could be released during the inflammatory response, which subsequently activated and recruited neutrophils and macrophages to the infection sites [124]. Both TNFα and IL1β are produced by a variety of immune cells after the activation of the hosts’ PRRs by PAMPs or DAMPs, whose main role is to initiate inflammation [125,126]. An inflammatory response could induce a cytokine cascade, characterized by TNFα release, followed by IL1β and other cytokine secretions [122]. In recent decades, an increasing number of studies were conducted on MC-induced immune dysfunction, especially regarding inflammation. In an in vivo study, the splenic TNFα and IL1β levels showed a significant decrease in grass carp when exposed to MC-LR [127]. In an in vitro study, the transcription levels of *tnfα* and *il1β* in the pronephros cells of common carp exhibited a significant increase when exposed to cyanobacterial extract and pure MC-LR [73]. These results indicated that MC-LR could result in a disturbance in the production of cytokines and an inflammatory response, subsequently interfering with the fish immune function. In our previous study, the transcription levels and concentration of IL1β and TNFα showed a remarkable and simultaneous increase, which proved that MC-LR could result in an inflammatory response in zebrafish [83].

Toll-like receptor (TLR) signaling pathways can recognize exogenous substances and induce cytokine release during the nonspecific immune process [128]. Myeloid differentiation factor 88 (MyD88) plays a critical role in the inflammatory responses induced by TLRs [129]. The activation of TLRs/MyD88 can regulate the immune defense by promoting the production of downstream cytokines including TNFα and IL1β. Our previous study revealed that MC-LR could induce chronic inflammation through the TLR/MyD88 signaling pathway in zebrafish [83]. Generally, acute inflammation exerts a positive immune function within minutes when the organism faces exogenous factors [130]. However, the immunologic balance could be disturbed when the inflammation persists for weeks or longer, eventually resulting in tissue damage [131].

### 4.6. Apoptosis

Apoptosis plays a vital role in cell growth and development, which is mainly characterized by nuclear chromatin condensation, cell shrinkage, and apoptotic body formation [132,133]. Zhang et al. [85] demonstrated that MC-LR exposure could result in the apoptosis in the lymphocytes of *C. auratus*. Our previous study found that the number of apoptotic cells and the expression of apoptotic genes increased significantly in the zebrafish spleen when exposed to MC-LR [83]. Researchers believe that MC-LR exposure could induce ROS formation and lipid peroxidation, which eventually results in cell apoptosis [134]. Up until now, increasing evidence has confirmed that MC-induced apoptosis was tightly associated with the ROS generation [135]. Rymuszka and Adaszek [73] indicated that the cyanobacterial extracts can induce apoptosis in common carp, which was confirmed by the increase in caspase-3 and caspase-7 activities in lymphocytes. The endoplasmic reticulum pathway is also involved in the MC-induced apoptosis [136]. These results demonstrated that the apoptosis of lymphocytes and phagocytes could be one of the potential mechanisms of MC-induced immunotoxicity on fish.

## 5. Current Research Gaps and Future Directions

### 5.1. Adaptive Immunity

Fish mainly depend on the innate immunity in the early stage, while the adaptive immunity is less developed [58]. An increasing number of studies were carried out to evaluate the MC-induced innate immunity in fish, and the research related to adaptive immunity was usually neglected because of experiment difficulties. The adaptive immunity, including humoral and cellular immunity, normally starts with a specific antigen-antibody recognition [137,138]. The adaptive immunity normally takes days to develop when the body is invaded by pathogens [139]. The identification of the specific molecules allows for a faster and stronger adaptive immunity when exposed to the same pathogen. More studies should be conducted to focus on MC-induced adaptive immunity.

### 5.2. Multi-Omics Study

In consideration of the organisms’ complexities and the potential interactions between various environmental stressors or factors, classic techniques are far from adequate to explore the in-depth immunotoxicity involving MCs. High-throughput analytical technologies, including metabolomics, proteomics, and genomics, need to be organized and utilized to carry out toxicological studies [140,141]. Lin et al. [83] demonstrated that the regulatory pathways concerning signal transduction were differentially regulated in the zebrafish spleen via a transcriptomic analysis, and the TLR/MyD88 signaling pathway was significantly activated to induce chronic inflammation. An increasing number of studies are being conducted to assess MC-induced toxicity in fish by using the ‘omics’ methodology [78,142,143]. The underlying mechanisms of the MC-induced immunotoxicity through multi-omics studies must be elucidated by combining the immune regulation pathways with the classic immune indices. The results will allow us to have a better understanding of hazardous chemicals or materials.

### 5.3. Hormesis Phenomenon

The dose–response relationship is a basic concept in toxicological studies [144]. The word ‘hormesis’ is used to describe a biphasic variation tendency, which is characterized by a high dose inhibition and a low dose stimulation; this gradually became a key concept in biological sciences and played a critical role in environmental risk assessment [145]. In view of the specific dose–response phenomenon, it is extremely important to determine the concentration threshold of the dosage effects [146]. Several previous studies observed a hormesis phenomenon in MCs exposure tests. Rymuszka et al. [33] found that lymphocytes’ proliferation showed a significant increase when exposed to low-concentrations of MC-LR, but a significant decrease when exposed to high-concentrations of MC-LR. Similarly, low-dose cyanobacteria lyophilized powder resulted in increased lysozyme activity in crucian carp, while high-dose cyanobacteria lyophilized power led to results of the opposite trends [67]. In our former study, zebrafish immune systems exhibited a dualistic tendency when sub-chronically exposed to different concentrations of pure MC-LR [31]. Very few studies provide a comprehensive explanation of such biphasic consequences, and thus future studies should explore the inner-most mechanism.

### 5.4. Variants Other than MC-LR

MC-LR is the most distributed variant, followed by MC-RR and MC-YR from over 270 identified MCs variants [11,12,13,147]. For MC congeners, other than MC-LR, there are limited data concerning their toxicity and occurrence in the scientific literature [148]. Previous studies demonstrated that tilapia and goldfish IP injected with MC-RR developed nephritic oxidative stress [79,80]. Numerous variants such as MC-LF and MC-LW have more lipophilic compounds than MC-LR, which indicates that other variants can be more toxic than MC-LR. Higher concentrations of variants such as MC-YR and MC-LF were detected instead of MC-LR and MC-RR in field studies, which implies that the toxicity of the lesser-known minority MC congeners require more attention [149]. A realistic risk evaluation should be based on the toxicity contribution of other variants, not merely on MC-LR for future immunotoxicity studies, e.g., MC-YR, MC-LF, MC-LA, and the mixtures.

### 5.5. Microbial Pathogens in Aquatic Ecosystems

Various kinds of microbial pathogens exist in natural waters, which could pose a threat to fish. Cyanobacteria bloom and their mucilaginous layers can be treated as a cozy microenvironment for microbial pathogens, which pose a joint threat to fish immune function [12]. In a sub-chronic study, Palikova et al. [150] found that a single cyanobacterial biomass, or the presence of white spot disease (*Ichthyophthirius multifiliis*), created a stimulating effect on the immune response, while the combined group caused immunosuppression in common carp. Polikova et al. [151] demonstrated that a cyanobacterial biomass enhanced the severity of the immune dysfunction of common carp injected with *Carp sprivivirus*, which indicated that the co-exposure to cyanobacteria and virus worsened the fish immunity. Such evidence proved that the synergistic effects of MCs and microbial pathogens should not be neglected.

## 6. Conclusions

**Figure 3:**
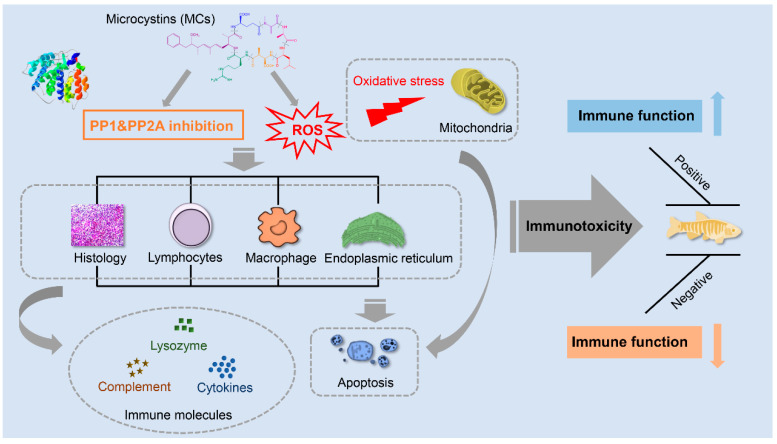
A schematic review of the immunotoxicity of MCs on fish. MCs are specific inhibitors of serine/threonine PP1 and PP2A, which result in the imbalance of phosphorylation and dephosphorylation of crucial proteins, subsequently causing abnormal cellular proliferation and cytoskeleton organization damages. MCs exposure is also related to the ROS overproduction and oxidative stress in immune organs, which results in apoptosis and immune dysfunction. MC-induced immunotoxicity greatly depends on exposure routes, time, and concentrations/doses.

As the immune system plays a vital role in maintaining a balance between MC-induced toxicity and an organisms’ survival strategy, more attention must be paid to the immune response when facing an exogenous threat. We made a comprehensive review compiled of existing research concerning MC-induced immunotoxicity on fish. These studies exhibited a tight association between immunotoxicity and MCs exposure doses and routes. MCs can induce an inflammatory response and lead to immune dysfunction by impairing immunocytes and influencing the secretion of immune molecules. The possible mechanisms of MC-induced immunotoxicity are summarized in Figure 3, implying that MC exposure poses a critical threat to the immune function of fish. Moreover, our recent study found that the parental exposure to MC-LR could interfere with the immune function of F1 larvae, which indicated that MCs could result in cross-generational immunotoxicity [152]. The alterations in immune parameters can be treated as reliable indicators to assess the health of fish following MCs exposure. Investigating the immunotoxicity and the underlying mechanisms of MCs is not only crucial for aquatic environment evaluation, but also for the sustainable development of fish populations in the future.

## Figures and Tables

**Table 1:** Summary of immunotoxicity of cyanobacterial extracts in fish studies in vivo ^a^.

Test Objects	Toxicant	Exposure	Doses/Concentrations	Time Points	Biological Responses	References
Nile tilapia	Cyanobacterial cells	Orally	120 μg MC-LR/fish	24 h	Kidney: ultrastructural damages, LPO ↑, GSH/GSSG ratio ↓, CAT ↓, SOD ↓, GR ↑, GPx ↑, GST ↓	[66]
Crucian carp	Cyanobacteria lyophilized powder	Orally	20% and 40% of cyanobacteria (1.41 mg/g MCs)	30 d	Spleen and head kidney: histopathological damages, macrophage bactericidal activity ↑, lysozyme activity ↑↓, blood nitroblue tetrazolium activity ↑	[67]
Blunt snout bream	Cyanobacteria lyophilized powder	Orally	1.41 mg/g MCs (Dry weight)	30 d	Head kidney: ultrastructural damages, white blood cells numbers↓, phagocytosis activity ↓, sIgM ↓, sIgD ↓, sIgZ ↓	[68]
Carp	Cyanobacterial cells	Immersion	5.6 × 10^4^–3.2 × 10^5^; 2.6 × 10^5^–3.6 × 10^6^ cells/mL	96, 168 h	Plasma: LDH ↑, TP ↑, ALT ↑, AST ↑	[69]
Silver carp	Cyanobacterial cells	Immersion	2.8–7.4 μg/L MCs	7, 14, 21, 28 d	Plasma: ALB ↓, ALP ↓, CHOL ↓, TP ↓, CRE ↓, LACT ↓, LDH ↓, P ↓, Fe ↓, CHE ↓, ALT ↑	[70]
Silver carp	Toxic *Microcystis* blooms	Immersion	0–15.58 μg/L MCs (averaged 4.16 μg/L)	Monthly (1 year)	Kidney: ultrastructural damages, CAT ↑, GST ↑, GSH ↓	[71]
Crucian Carp	Cyanobacteria extract	IP injection	50, 200 μg MC-LR equiv kg^−1^ BW	12, 24, 48, 60 h	Plasma: ALT ↑, ALP ↑, AST ↑, LDH ↑, GLU ↑↓, CHO ↓, TG ↓, TP ↓	[72]
Common Carp	Cyanobacteria extract	Immersion	25 μg/L MCs	1, 3, 5 d	Blood and head kidney: Intracellular O^2−^ production ↑, ROS ↑, lymphocyte proliferation ↓, IL-1β ↑, TNF-α ↑, IL-10 ↑	[73]
Common carp	Cyanobacteria extract	Immersion	1.3 μg/L, 13 μg/L MCs	8, 30 d	Blood: hematocrit value ↑, hemoglobin concentration ↑, phagocytic activity ↓, total plasma protein ↑, AST ↓, LDH ↓	[74]

^a^ ↑ indicates activation, increasing or upregulation; ↓ indicates inhibition, decreasing or downregulation.

**Table 2:** Summary of immunotoxicity of pure microcystin in fish studies in vivo ^a^.

Test Objects	Toxicant	Exposure	Doses/Concentrations	Time Points	Biological Responses	References
Silver carp.	MC-LR	IP	104.9 μg/kg, 262.1 μg/kg	6, 9, 12, 24, 72, 168 h	Serum: ALT ↑, AST ↑, lysozyme activity ↑, complement C3 ↑, TNF-α ↑, IL-1β ↑, IFN-γ ↑	[75]
Common carp	MC-LR	IP	150 μg/kg BW	28 d	Serum: CAT ↑, SOD ↑, GSH ↑, GPx ↑, LPO ↑, complement C3 ↓, lysozyme activity ↓	[76]
Grass carp	MC-LR	IP	50 μg/kg	1, 2, 7, 14, 12 d	Spleen and head kidney: mitochondrial edema, chromatin condensation, apoptotic lymphocytes	[34]
Bighead carp	MC-LR	IP	50, 200, 500 μg MC-LR/kg BW	3, 24 h	Liver and kidney: temporal- and dose-dependent increase in interleukin-8	[77]
Grass carp	MC-LR	IP	25, 75, 100 μg/kg BW	96 h	Liver: complement and coagulation cascades pathway ↑, *soc3* ↑, *hsp70* ↑,	[78]
Gold fish	MC-RR	IP	50, 200 μg/kg BW	6, 12, 24, 48 h	Kidney: T-AOC ↓, SOD ↑, GPx ↓	[79]
Tilapia	MC-LR, -RR	IP	500 μg/kg MC-LR or MC-RR	7 d	Kidney: SOD ↑, CAT ↑, GPx ↑, LPO ↑,	[80]
Zebrafish	MC-LR	Immersion	200, 800 μg/L	12, 24, 48, 96, 168 h	Larvae: Rag1 ↑, Rag2 ↑, Ikaros ↑, GATA1↑, Lck↑, TCRα↑	[81]
Zebrafish	MC-LR	Immersion	0, 1, 5, 20 μg/L	30 d	Spleen: ultrastructural damages, *ifn1* ↑, *il8* ↑, *il1β* ↑, *tnfα* ↑	[82]
Zebrafish	MC-LR	Immersion	0.3, 1, 3, 10, 30 μg/L	30 d	Spleen: histopathological lesions, complement C3 ↑↓;	[31]
Zebrafish	MC-LR	Immersion	0, 0.4, 2, 10 μg/L	30 d	Spleen: histopathological lesions, apoptosis, TNF-α ↑, IL-1β ↑, MYD88 ↑, complement C3 ↑↓	[83]
Zebrafish	MC-LR	Immersion	20 μg/L	14 d	Liver: TBARS ↑, GSH ↑, LDH ↑, GST ↓, CAT ↓, *cas3a* ↑, *cas3b* ↑	[84]

^a^ ↑ indicates activation, increasing or upregulation; ↓ indicates inhibition, decreasing or downregulation.

**Table 3:** Summary of immunotoxicity of pure microcystins in fish studies in vitro ^a^.

Test Objects	Toxicant	Doses/Concentrations	Time Points	BiologicalResponses	References
Crucian carp lymphocytes	MC-LR, MC-RR	1, 5, 10 nM	2, 4, 6, 8 h	Apoptosis, nuclear chromatin condensation	[85]
Crucian carp lymphocytes	MC-LR	10 nM	0.5, 1, 3, 6 h	Apoptosis, intracellular Ca^2+^ ↑, ROS ↑, MMP↓, ATP ↓,	[86]
Crucian carp lymphocytes	MC-RR	10 nM	0.25, 0.5, 1, 3, 6 h	Apoptosis, MMP ↓, ROS ↑, intercellular ATP ↓	[87]
Crucian carp lymphocytes	MC-LR	1 μg/L	24 h	Apoptosis, MMP ↓, ROS ↑, GSH ↓, SOD ↓, CAT ↓, MDA ↑	[88]
Rainbow trout lymphocytes	MC-LR	1, 5, 10, 20, 40 μg/mL	4, 24, 48, 72, 96, 120 h	Cell viability ↓, lymphocytes proliferation ↑↓	[33]
Rainbow trout phagocytic cells	MC-LR	1, 5, 10, 20 μg/mL	2, 4, 24 h	Time- and concentration-dependent cell viability decrease, phagocytic cell ability ↑↓, respiratory burst activity ↑↓	[89]
CIK cells	MC-LR	1, 10, 100 μg/L	24, 48 h	Apoptosis, cytoskeleton disruption, cell viability ↑↓, ROS ↑, MDA ↑, GSH ↓, GST ↑, SOD ↓	[90]
Carp leucocytes	MC-LR	0.01, 0.1, 0.5, 1 μg/mL	24, 72 h	Respiratory burst activity ↑↓, B lymphocytes proliferation ↑, necrosis of leucocytes ↑	[91]
Common carp lymphocytes and phagocytes	MC-LR	0.01, 0.05, 0.1, 1 μg/mL	2, 6, 24 h	Phagocytosis ↓, LDH ↑, GSH ↓, apoptosis, necrosis	[92]
Common carp leukocytes	MC-LR	0.01, 0.1 μg/mL	4 h	*il1β* ↑↓, *tnfα* ↑↓, *il10* ↑, *tgfβ* ↑	[93]

^a^ ↑ indicates activation, increasing or upregulation; ↓ indicates inhibition, decreasing or downregulation.
